# Identification and validation of NAD+ metabolism-related biomarkers in patients with diabetic peripheral neuropathy

**DOI:** 10.3389/fendo.2024.1309917

**Published:** 2024-02-23

**Authors:** Chenhao Ye, Yuedong Fu, Xijie Zhou, Feiya Zhou, Xuwei Zhu, Yiheng Chen

**Affiliations:** Department of Hand and Microsurgery, The Second Affiliated Hospital and Yuying Children’s Hospital of Wenzhou Medical University, Wenzhou, China

**Keywords:** nicotinamide adenine dinucleotide metabolism, bioinformatic analysis, biomarkers, gene expression, diabetic peripheral neuropathy

## Abstract

**Background:**

The mechanism of Nicotinamide Adenine Dinucleotide (NAD+) metabolism-related genes (NMRGs) in diabetic peripheral neuropathy (DPN) is unclear. This study aimed to find new NMRGs biomarkers in DPN.

**Methods:**

DPN related datasets GSE95849 and GSE185011 were acquired from the Gene Expression Omnibus (GEO) database. 51 NMRGs were collected from a previous article. To explore NMRGs expression in DPN and control samples, differential expression analysis was completed in GSE95849 to obtain differentially expressed genes (DEGs), and the intersection of DEGs and NMRGs was regarded as DE-NMRGs. Next, a protein-protein interaction (PPI) network based on DE-NMRGs was constructed and biomarkers were screened by eight algorithms. Additionally, Gene Set Enrichment Analysis (GSEA) enrichment analysis was completed, biomarker-based column line graphs were constructed, lncRNA-miRNA-mRNA and competing endogenouse (ce) RNA networks were constructed, and drug prediction was completed. Finally, biomarkers expression validation was completed in GSE95849 and GSE185011.

**Results:**

5217 DEGs were obtained from GSE95849 and 21 overlapping genes of DEGs and NMRGs were DE-NMRGs. Functional enrichment analysis revealed that DE-NMRGs were associated with glycosyl compound metabolic process. The PPI network contained 93 protein-interaction pairs and 21 nodes, with strong interactions between NMNAT1 and NAMPT, NADK and NMNAT3, ENPP3 and NUDT12 as biomarkers based on 8 algorithms. Expression validation suggested that ENPP3 and NUDT12 were upregulated in DPN samples (P < 0.05). Moreover, an alignment diagram with good diagnostic efficacy based on ENPP3 and NUDT12 were identified was constructed. GSEA suggested that ENPP3 was enriched in Toll like receptor (TLR) pathway, NUDT12 was enriched in maturity onset diabetes of the young and insulin pathway. Furthermore, 18 potential miRNAs and 36 Transcription factors (TFs) were predicted and the miRNA-mRNA-TF networks were constructed, suggesting that ENPP3 might regulate hsa-miR-34a-5p by affecting MYNN. The ceRNA network suggested that XLOC_013024 might regulate hsa-let-7b-5p by affecting NUDT12. 15 drugs were predicted, with 8 drugs affecting NUDT12 such as resveratrol, and 13 drugs affecting ENPP3 such as troglitazone.

**Conclusion:**

ENPP3 and NUDT12 might play key roles in DPN, which provides reference for further research on DPN.

## 1 Introduction

Diabetic peripheral neuropathy (DPN) is a common complication that occurs in individuals with diabetes and is closely associated with detrimental effects, particularly in the context of diabetic foot. The diminished sensation caused by DPN can prevent timely detection of external stimuli and pressure, resulting in a heightened vulnerability to injuries and wounds (1). DPN can lead to severe consequences, including an increased risk of foot ulcers, infections and even lower extremity amputations (1). Additionally, DPN-induced excruciating pain significantly impacts the patients’ quality of life, causing persistent discomfort during sleep, walking and daily activities (2, 3). Furthermore, the understanding of the underlying mechanisms of DPN remains incomplete (4), and the available treatment options are limited, primarily focused on symptom management (5). Therefore, it is crucial to delve deeper into the mechanisms of diabetic peripheral neuropathy and develop novel approaches for early detection and intervention, given the current challenges in effectively treating DPN.

Nicotinamide Adenine Dinucleotide (NAD+) plays a critical role in cellular energy metabolism and redox reactions (6). It serves as an essential coenzyme for numerous enzymatic reactions involved in glycolysis, the tricarboxylic acid cycle, and oxidative phosphorylation (7). By accepting and donating electrons, NAD+ participates in vital cellular processes such as ATP production, DNA repair and gene expression regulation (8). NAD+ is also an essential cofactor for non-redox NAD+-dependent enzymes, including sirtuins and poly(ADP-ribose) polymerases (PARPs) (9), which are involved in cellular homeostasis and stress responses (10). Recent studies have indicated that NAD+ exerted an influence on the SIRT1-PGC-1α-TFAM pathway, contributing to the occurrence of DPN (11, 12). Nicotinamide riboside (NR) is a precursor of the NAD salvage pathway that enhances respiratory function, reduces mitochondrial ROS (mtROS) production, and reduces IL-1B production in peripheral blood mononuclear cells (PBMCs) (13). In addition, studies have shown that the increase of NAD levels in heart failure is associated with the improvement of maximum mitochondrial respiration of PBMC and the decrease of the expression of pro-inflammatory markers of PBMC, that is, the increase of NAD levels has potential beneficial effects on mitochondrial function and inflammatory activation (14). These observations raise the possibility that DPN patients may benefit by regulating genes associated with NAD+ metabolism. Future discoveries on disease pathogenesis will be crucial to successfully address all aspects of DPN, from prevention to treatment (5). The combined study of DPN and NAD+ metabolism-related genes (NMRGs) is an area that has received limited attention in current research (15). However, investigating the intricate relationship between DPN and NMRGs holds significant scientific value. By integrating the analysis of DPN pathogenesis and the influence of NMRGs on NAD+ metabolism, we can gain deeper insights into the underlying molecular mechanisms. This integrated approach has the potential to uncover novel biomarkers, therapeutic targets, and personalized treatment strategies for DPN. Therefore, further exploration in this field is crucial to enhance our understanding of DPN and bridge the gap between basic research and clinical applications, ultimately improving patient outcomes.

In the genomic era, gene chips have been widely used to explore disease mechanisms, providing new insights into the pathogenesis at the genetic level (16). Therefore, this study aims to identify and validate NAD+ metabolism-related biomarkers in patients with DPN using bioinformatics analysis and provide a reference for further DPN research.

## 2 Materials and methods

### 2.1 Data source

Enter keywords Diabetic Peripheral Neuropathy into the Gene Expression Omnibus (GEO) database (https://www.ncbi.nlm.nih.gov/gds) to determine the species as Homo sapiens. In order to ensure the reliability of the data, more than 5 disease and control samples were controlled to obtain our dataset, and two DPN datasets (GSE95849 and GSE185011) were acquired. The GSE95849 includes gene expression data for 6 peripheral blood mononuclear cell (PBMC) samples of DPN patients and 6 PBMC samples for normal control. The GSE185011 includes gene expression data for 5 PBMC samples of DPN patients and 5 PBMC samples for normal control. A total of 51 NMRGs were collected from previous article (17).

### 2.2 Differential expression analysis

Differential expression analysis was performed between DPN samples and control samples using the limma R package (18) in the GSE95849 to screen differentially expressed genes (DEGs) by setting |log fold change (FC)| > 1 and adjust *P*-values (*P*.adj) < 0.05. To better visualize the differences in gene expression among the two groups, the R packages ggpubr and ggplot2 were used to plot volcano and heat maps of DEGs, respectively. The intersecting genes of DEGs and NMRGs were obtained with R package ggvenn and defined as DE-NMRGs. In addition, Gene ontology (GO) and Kyoto encyclopedia of genes and genomes (KEGG) enrichment analysis of DE-NMRGs were completed using the clusterProfiler package (19) to explore the functions of DE-NMRGs.

### 2.3 Acquisition of biomarkers

To explore whether protein interactions existed between the DE-NMRGs, a protein-protein interactions (PPI) network was created by STRING (https://string-db.org) database. Topological properties of PPI network nodes were analyzed using eight algorithms (Degree, Density of Maximum Neighborhood Component (DMNC), Edge Percolated Component (EPC), Maximal Clique Centrality (MCC), Maximum Neighborhood Component (MNC), Closeness, Radiality, Clustering Coefficient (CC)) in Cytohubba plugin, and the TOP 10 genes from each algorithm were selected for taking intersection using UpsetR (20) R package according to the ranking, the intersection genes were defined as biomarkers. Expression validation of biomarkers was completed in GSE95849 and gse185011. In order to understand whether there was a correlation among biomarkers, correlations among the biomarkers were calculated. In addition, the analysis of regulatory relationships among biomarkers and their interacting genes as well as the enrichment analysis was conducted with the GeneMANIA database (http://genemania.org).

### 2.4 Construction of alignment diagram and gene set enrichment analysis

Alignment diagram of biomarkers were constructed using the rms package (21) in R. The predictive power of the alignment diagram was assessed using calibration curves and decision curves. Moreover, to explore the potential mechanism of biomarkers, Gene Set Enrichment Analysis (GSEA) was conducted with ClusterProfiler package (19) in GSE95849, the KEGG pathway gene set and GO biological process gene set were used as the enrichment background. The significant enrichment threshold was set as |Normalized Enrichment Score (NES)|>1& nominal *P*-value (NOM *P*-val) <0.05. Biomarkers-related diseases were predicted using the Comparative Toxicogenomics Database (CTD, http://ctdbase.org/) based on Inference Score > 60 and the biomarker-diseases network was visualized using Cytoscape software.

### 2.5 Construction of a miRNA-mRNA-TF regulatory network and ceRNA network and drug prediction

To understand the regulatory relationships associated with biomarkers, potential target miRNAs of biomarkers were predicted by the miRNet database (https://www.mirnet.ca/) and NetworkAnalyst database (https://www.networkanalyst.ca/). The prediction results of the above two databases were intersected to obtain the intersection miRNA. In addition. Moreover, the potential transcription factors (TF) of biomarkers were predicted by the NetworkAnalyst database. The miRNA-mRNA-TF interaction network was constructed using Cytoscape. Moreover, the possibly regulated lncRNAs of intersection miRNAs were retrieved in the lncBaseV2 database (http://carolina.imis.athena-innovation.gr/diana_tools/web/index.php), and lncRNA-miRNA-mRNA network was constructed using Cytoscape. Drugs that may have potential effects on biomarkers were predicted by using the CTD (http://ctdbase.org/), and to visualize the relationship among genes and drugs, gene-drugs networks were constructed by Cytoscape.

### 2.6 Statistical analysis

Limma was used to identify DEGs. The Benjamini & Hochberg (BH) test was used for multiple test correction screening of DEGs. Venn and UpsetR was used for multiple gene sets to take intersections. The associations among the genes determined using the spearman correlation analysis. Statistical analysis was carried out through R software (version 4.1.1, https://www.r-project.org/). Differences between groups were analyzed via the Wilcox test. P < 0.05 represented a significant difference.

## 3 Results

### 3.1 DE-NMRGs were associated with glycosyl compound metabolic process

**Figure 1:**
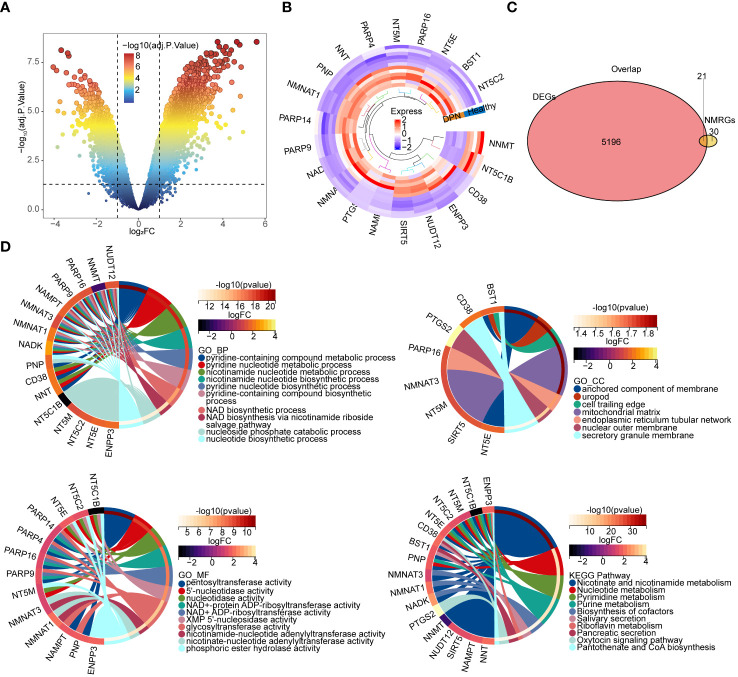
Identification and enrichment analysis of DE-NMRGs. **(A)** Volcano plot of differentially expressed genes. The upper-left nodes represent downregulated DEGs, the upper-right nodes represent upregulated DEGs. **(B)** Heat map and clustering analysis DEGs and NMRGs. The marked genes are the hub genes obtained by subsequent analysis. **(C)** Venn diagram of shared gene between DEGs. DEGs: Differentially expressed genes screened from GSE95849. NMRGs: NAD+ metabolism related genes downloaded from previous article (17) **(D)** Chord diagram of the top 10 enriched terms of DE-NMRGs. The upper left plot is BP enrichment; the upper right plot is CC enrichment; the lower left plot is MF enrichment; the lower right plot is KEGG pathway enrichment analysis chord diagram. BP, Biological Process; CC, Cellular Component; MF, Molecular Function.

A total of 5217 DEGs were obtained from the GSE95849, and 3691 DEGs were upregulated and 1526 DEGs were downregulated in DPN (**Figures 1A, B**). A total of 21 overlapping genes of DEGs and NMRGs were defined as DE-NMRGs (**Figure 1C**). Moreover, functional enrichment analysis revealed that DE-NMRGs were associated with the nicotinate and nicotinamide metabolism, NAD biosynthesis via nicotinamide riboside salvage pathway and other functional pathways related to NAD metabolism, enriched in pyridine-containing compound metabolic process and pyridine nucleotide metabolic process in GO terms, and enriched in NAD biosynthetic process and glycosyl compound metabolic processin KEGG terms (**Figure 1D**).

### 3.2 Two biomarkers were obtained by using machine learning algorithms

**Figure 2:**
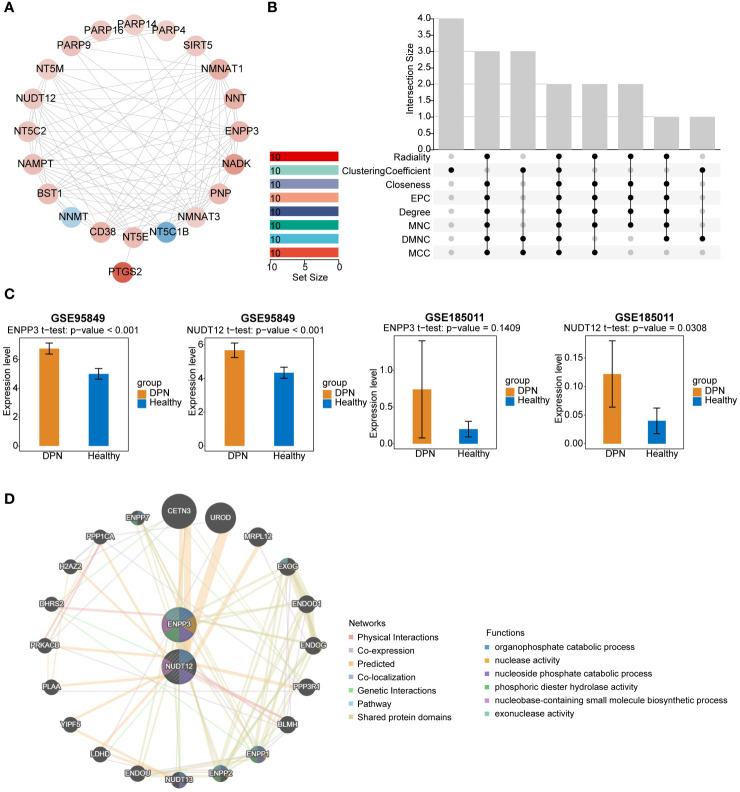
PPI network analysis and validation of potential biomarkers. **(A)** PPI network diagram of DE-NMRGs. Red indicates genes that are up-regulated, whereas blue indicates genes that are down-regulated. **(B)** Venn diagram showing the overlap of potential biomarkers were identified by 8 different algorithms. **(C)** Validation of ENPP3 and NUDT12 expression in GSE95849 and GSE185011. **(D)** PPI regulatory network of ENPP3 and NUDT12.

To explore whether protein interactions existed among these 21 DE-NMRGs, a PPI network was created (**Figure 2A**) by setting a confidence level as 0.4 (Confidence = 0.4). There were 93 protein-interaction relationship pairs and 21 nodes, with strong interactions between NMNAT1 and NAMPT, NADK and NMNAT3. Topological properties of PPI network nodes were analyzed (**Figure 2B**), ENPP3 and NUDT12 were identified as biomarkers based on 8 algorithms. Expression validation of biomarkers was completed in GSE95849 and GSE185011. The results of expression validation suggested that NUDT12 showed significant differences in both training and validation sets. Alteration of ENPP3 expression was not significant in the validation set, however the trend of biomarker ENPP3 remained consistent in both data sets, and both biomarkers showed upregulation in the disease samples (**Figure 2C**). The PPI regulatory network of biomarkers constructed by GeneMANIA database was shown in the **Figure 2D**, suggesting that biomarkers were enriched to pathways such as organophosphate catabolic process, nucleoside phosphate catabolic process.

### 3.3 Alignment diagram with good diagnostic efficacy based on biomarkers were constructed

**Figure 3:**
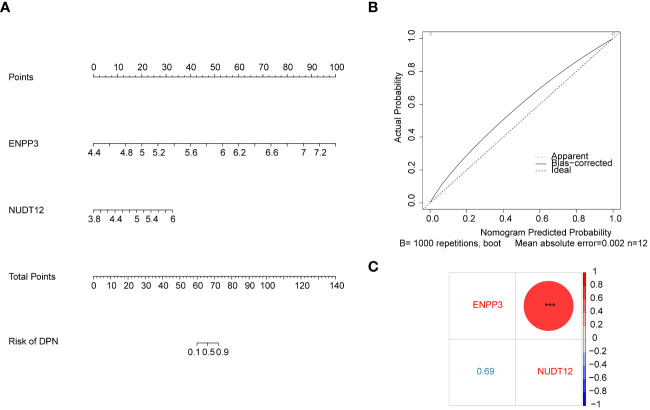
Diagnostic model construction and correlation analysis based on biomarkers. **(A)** Nomogram based on the expression of biomarkers ENPP3 and NUDT12. **(B)** Calibration curve of the nomogram model in **(A)** The x-axis shows the nomogram-predicted probability of DPN. The y-axis shows the actual probability of DPN occurrence **(C)** Bubble chart showing correlation analysis between the expression levels of biomarkers ENPP3 and NUDT12.

Moreover, based on the biomarkers, an alignment diagram was constructed, and the score of each sample was calculated by the alignment diagram, the higher the total score of the patient, the higher the likelihood that the patient will develop DPN disease (**Figure 3A**). The calibration curve suggested that the alignment diagram possesses good diagnostic efficacy (**Figure 3B**). Correlation analysis among biomarkers suggested a significant positive correlation (**Figure 3C**).

**Figure 4:**
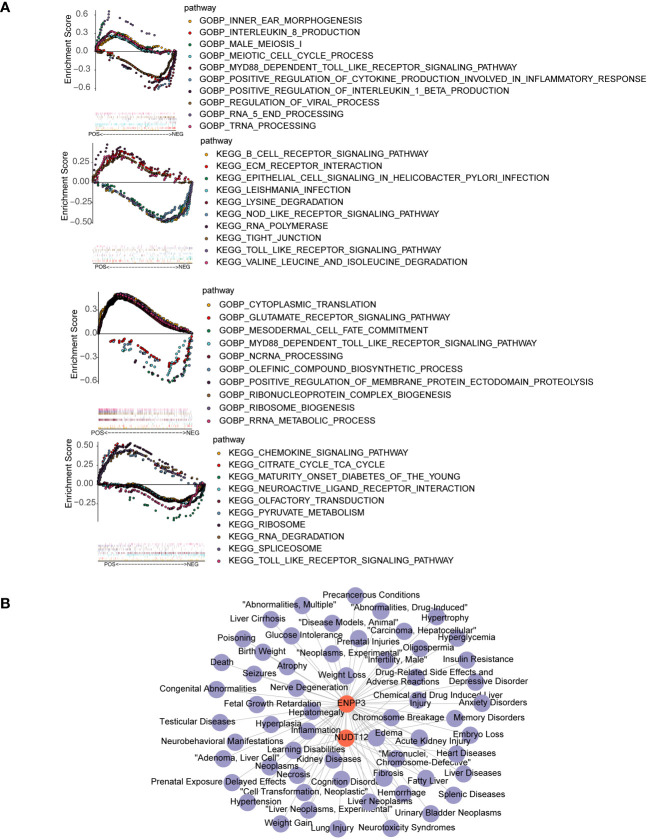
GSEA enrichment and disease association analysis of biomarkers. **(A)** GSEA enrichment results for biomarkers ENPP3 (upper) and NUDT12 (lower). The left figures show top 10 enriched GO biological processes (BP); the right figures show top 10 enriched KEGG pathways. **(B)** Biomarker-disease association network. Red nodes represent biomarkers and purple nodes represent associated disease.

The results of GSEA suggested that the ENPP3 was enriched in Toll like receptor signaling pathway and natural killer cell mediated cytotoxicity, NUDT12 was enriched in maturity onset diabetes of the young and insulin signaling pathway (**Figure 4A**). A total of 59 biomarker-associated diseases were obtained through the CTD database, and a total of 55 biomarker-disease association pairs were obtained, such as ENPP3 and Glucose Intolerance, and NUDT12 and Neurotoxicity Syndromes (**Figure 4B**).

### 3.4 Biomarkers were associated with oxidative phosphorylation

**Figure 5:**
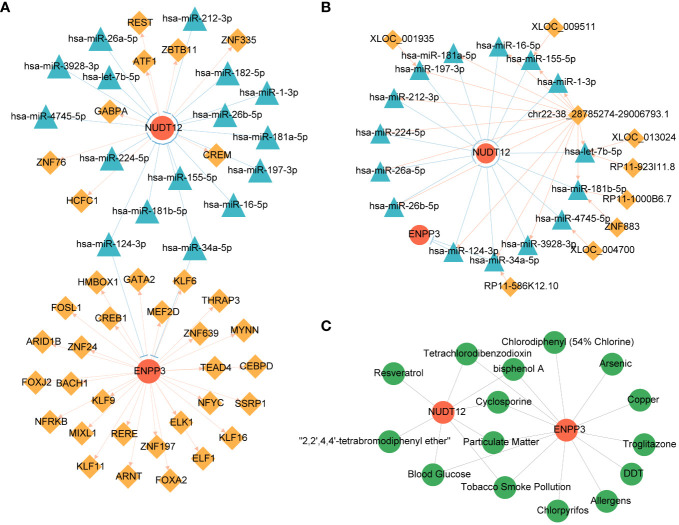
Regulatory network and drug prediction analysis of biomarkers. **(A)** miRNA-mRNA-TF regulatory network of biomarkers. Red nodes represent biomarkers; yellow diamonds TFs; blue triangles represent miRNAs. **(B)** ceRNA network of biomarkers. Red nodes represent biomarkers; yellow diamonds represent lncRNAs; blue triangles represent miRNAs. **(C)** Drug-target network of biomarkers. Red nodes represent biomarkers; green nodes represent drugs.

A total of 18 potential miRNAs and 36 TF were predicted and the miRNA-mRNA-TF interaction networks were constructed, the network suggested that ENPP3 was regulated by hsa-miR-34a-5p and MYNN (**Figure 5A**). A total of 25 lncRNA-miRNA-mRNA relationship pairs were obtained, and ceRNA networks were constructed which contains 9 lncRNA and 15 miRNA, the network suggested that XLOC_013024 might regulate NUDT12 by affecting hsa-let-7b-5p (**Figure 5B**). A total of 15 drugs were predicted, among them, 8 drugs had potential effects on NUDT12 such as resveratrol, and 13 drugs had potential effects on ENPP3 such as troglitazone (**Figure 5C**).

## 4 Discussion

After our comprehensive analysis, 5217 DEGs were obtained, including 21 DE-NMRGs, shedding light on their role in DPN. A key finding is the identification of NUDT12 and ENPP3 as central biomarkers in these DEGs, offering novel insights into DPN’s pathogenesis. Our bioinformatics analysis indicates these genes are upregulated in DPN, suggesting their potential impact on disease progression. NUDT12, involved in NAD+ metabolism and associated insulin signaling and diabetic pathways, along with ENPP3, linked to purinergic signaling and inflammatory responses, both contribute critically to metabolic and inflammatory aspects of DPN.

Neuronal mitochondrial(Mt) dysfunction is an important mechanism leading to axonal degeneration in DPN (22). As a result of Mt damage, oxidative stress occurs in diabetic dorsal root ganglion (DRG) neurons, axons, and Schwann cells, and has been proposed as a unifying mechanism for diabetic neuropathy (12, 23). One of the key pathways that is impaired in DPN is the energy sensing pathway comprising the NAD+-Dependent SIRT1–PGC-1α–TFAM pathway (12). The mitochondrial energy metabolism is greatly dependent on NAD+ as a cofactor that is essential for both the activity of respiratory and TCA cycle enzymes (24). NAD+ and its precursors play critical roles in DPN progression by modulating SIRT1 activity. Administration of NAD+ precursors nicotinamide riboside (NR) and nicotinamide mononucleotide (NMN) could prevent and reverse DPN in animal models, indicating that correct NAD+ depletion in the DRG may be sufficient to prevent DPN (23, 24). However, we suggest that the mere supplementation of NAD+ precursors may not suffice for disease modification. An in-depth understanding of the roles of NMRGs could provide key insights into the complex molecular mechanisms of DPN. Herein, we identified 21 DE-NMRGs. The enrichment analysis revealed that DE-NMRGs were primarily engaged in nicotinate and nicotinamide metabolism, NAD biosynthesis via nicotinamide riboside salvage pathway and other functional pathways related to NAD metabolism. The topological properties of these genes were analyzed by algorithm to obtain two biomarkers NUDT12 and ENPP3.

NUDT12 is a Protein Coding gene (25). Among its related pathways are Metabolism of water-soluble vitamins and cofactors and Nicotinate metabolism (25). The human NUDT12 Nudix hydrolase has been expressed in insect cells from a baculovirus vector as a His-tagged recombinant protein (26, 27). *In vitro*, it efficiently hydrolyses NAD(P)H to NMNH (the reduced-form of NMN) and AMP (2’,5’-ADP), and diadenosine diphosphate to AMP. It also has activity towards NAD(+), ADP-ribose and diadenosine triphosphate (26). For NUDT12, some previous studies have linked its overexpression with oxidative stress and redox imbalance in neural tissues, while others argued the association was circumstantial (25, 26, 28). Our results not only validated the upregulated expression of NUDT12, but also revealed its involvement in insulin signaling and diabetes-related pathways through enrichment analysis. The results showed NUDT12 is enriched in maturity onset diabetes of the young (MODY), a common form of monogenic type 2 diabetes in youth (29). Advances in molecular diagnostics have identified 14 different subtypes of MODY. Specially, insulin secretion defect is the molecular pathogenesis of MODY10, MODY11, MODY12, MODY13, MODY14.The insulin ligation enhances neurite outgrowth by activating the PI3K–Akt signaling pathway and attenuates phenotypic features of DPN (30). In addition, Insulin signaling plays an important role in restoring the myelin proteins in DPN (31). Given the involvement of NUDT12 in NAD+ metabolism, supporting that it may impact DPN by influencing insulin sensitivity and glucose homeostasis. These novel findings suggest NUDT12 may contribute to DPN pathogenesis by disrupting insulin signaling and glucose homeostasis, although the specific mechanisms need further validation.

On the other hand, ENPP3 has gained wide attention recently as a purinergic signaling-related gene. Among its related pathways are metabolism of water-soluble vitamins and cofactors and NAD metabolism (32). ENPP3 is involved in various physiological and pathological processes, such as nucleotide and phospholipid signaling, bone mineralization, fibrotic diseases and tumor-associated immune cell infiltration. ENPP3 also limits the inflammatory and allergic responses of mast cells and basophils, by eliminating extracellular ATP as a signaling molecule, inhibiting the activation and pro-inflammatory cytokine release of mast cells and basophils (33). According to the results, ENPP3 is enriched in the Toll like receptor (TLR) signaling pathway. TLRs can recognize pathogen-associated molecular patterns and activate downstream nuclear factor κB (NF-κB) to induce inflammatory cytokine release and inflammatory responses (34). ENPP3 may influence TLR activation by regulating extracellular ATP levels, and inflammation is also an important pathogenic mechanism of DPN. As a member of ectonucleotide pyrophosphatase family, studies have shown ENPP3 could modulate neuronal metabolism and purinergic signaling by regulating ATP levels (35, 36). ATP acts as a neurotransmitter and modulator of neurotransmitter release, but also as a trophic factor that stimulates proliferation and differentiation of neural cells (37). Purinergic signaling also plays important roles in neuronal function and diabetes. Extracellular ATP can activate P2X and P2Y receptors, causing downstream calcium influx and signal transduction (38). This disturbance of neuronal homeostasis can render neurons more vulnerable to damage in diabetes. Further investigation into the precise molecular mechanisms connecting ENPP3, purinergic signaling, calcium signaling and DPN development is still needed.

This study found that the competitive endogenous RNA network showed that XLOC_013024 may regulate NUDT12 by inhibiting hsa-let-7b-5p. NUDT12 is a Nudix hydrolase involved in NAD+ metabolism. As mentioned before, NUDT12 is associated with MODY, supporting its potential role in influencing diabetic peripheral neuropathy through affecting insulin and glucose homeostasis. lncRNAs and miRNAs can reciprocally regulate each other and form competitive endogenous RNA networks to control gene expression. Therefore, this finding suggests that XLOC_013024 and hsa-let-7b-5p may participate in the occurrence of diabetic peripheral neuropathy through NUDT12. XLOC_013024 may act as a competitive RNA to inhibit hsa-let-7b-5p, while NUDT12 is a downstream target of hsa-let-7b-5p. Abnormal XLOC_013024 may lead to altered activity of hsa-let-7b-5p, which then affects diabetic peripheral neuropathy through NUDT12-mediated pathways, but this network still needs experimental verification. In addition, this study also found that ENPP3 may regulate the expression of hsa-miR-34a-5p by affecting the transcription factor MYNN. As a nucleotidase regulating ATP levels, ENPP3 may affect metabolism by influencing neuronal response to ATP. Ghada Tagorti et al. found hsa-miR-34a-5p interacts with key genes related to type 2 diabetes (39). MYNN is involved in gene expression regulation of various biological processes. Therefore, we speculate that increased expression of ENPP3 may affect MYNN activity, dysfunction of MYNN may in turn lead to altered miRNA expression such as hsa-miR-34a-5p, eventually resulting in neuronal dysfunction. This newly discovered regulatory axis provides new insights into exploring the key pathological processes of diabetic peripheral neuropathy. However, the exact molecular mechanisms and functional significance of these regulatory networks still require experimental validation.

We predicted potential drugs that may affect the identified biomarkers using the CTD. The results showed that resveratrol was predicted to target NUDT12. Resveratrol is a natural polyphenol enriched in foods such as red grapes and red wine. It has been widely studied for its antioxidative, anti-inflammatory, antidiabetic and neuroprotective properties (40). For example, a previous study showed that resveratrol could alleviate diabetic peripheral neuropathy in mice by activating Nrf2 and inhibiting NF-kB pathways (41). It also reduced oxidative stress and improved Sirtuin 1 level in elderly patients with type 2 diabetes (42). Additionally, a systematic review suggested resveratrol could lower blood glucose and improve insulin resistance in adults with type 2 diabetes (43). These multifaceted effects indicate resveratrol may participate in the pathogenesis of diabetes and its complications through modulating oxidative stress, inflammation, glycemic control and more. Moreover, troglitazone was predicted to target ENPP3. Troglitazone acts as a free-radical scavenger and TNF-α inhibitor, can inhibit the slowing of motor nerve conduction velocity and morphological changes of the peripheral nerve in diabetic rats. troglitazone has a beneficial effect on peripheral neuropathy in streptozotocin-induced diabetic rats irrespective of blood glucose concentrations (44). In addition, there are some drugs that target NUDT12 and ENPP3 at the same time, such as Tetrachlorodibenzodioxin, cyclosporine, etc. However, its exact mechanisms of action still require further research. As a potential drug targetting NUDT12 and ENPP3 respectively predicted in this study, resveratrol and troglitazone may serve as a promising supplementary therapy for DPN pending experimental and clinical validation. Further studies are warranted to verify its efficacy and safety.

This study had certain limitations. Firstly, the sample size of patients used in this study was small, and we planned to expand the number of cohorts in future research. Secondly, this study only used bioinformatics to identify the NAD+ metabolism-related biomarkers associated with DPN, necessitating more biological experiments to validate the specific mechanisms of the biomarkers screened. Finally, there was potential for gene overlap between DPN patients and other vascular risk factors. Future research should emphasize larger-scale studies and experimental approaches to further elucidate the specific roles of these genes in DPN.

## 5 Conclusion

In conclusion, our comprehensive bioinformatics analysis has identified NUDT12 and ENPP3 as crucial biomarkers in DPN, emphasizing their roles in metabolic and inflammatory pathways linked to the disease, and constructed a diagnostic model based on the two genes. This study lays the foundation for elucidating the roles of the two genes in disease progression, and provides reference for developing personalized diagnosis and treatment of DPN. However, it is crucial to validate these findings through larger-scale research to further explore their clinical application value and expression mechanisms.

## Data availability statement

Publicly available datasets were analyzed in this study. This data can be found here: GEO database (https://www.ncbi.nlm.nih.gov/gds) STRING (https://string-db.org) database GeneMANIA database (http://genemania.org). Comparative Toxicogenomics Database (CTD, http://ctdbase.org/) miRNet database (https://www.mirnet.ca/) NetworkAnalyst database (https://www.networkanalyst.ca/) lncBaseV2 database (http://carolina.imis.athena-innovation.gr/diana_tools/web/index.php).

## Ethics statement

Ethical approval was not required for the study involving humans in accordance with the local legislation and institutional requirements. Written informed consent to participate in this study was not required from the participants or the participants’ legal guardians/next of kin in accordance with the national legislation and the institutional requirements.

## Author contributions

CY: Conceptualization, Data curation, Formal analysis, Investigation, Project administration, Resources, Software, Validation, Visualization, Writing – original draft. YF: Data curation, Formal analysis, Investigation, Project administration, Software, Supervision, Visualization, Writing – original draft. XiZ: Conceptualization, Formal analysis, Methodology, Project administration, Supervision, Writing – review & editing. FZ: Project administration, Writing – review & editing, Data curation, Investigation, Software, Validation. XuZ: Project administration, Software, Writing – review & editing, Conceptualization, Formal analysis, Methodology, Visualization. YC: Formal analysis, Methodology, Project administration, Visualization, Writing – review & editing, Data curation, Investigation, Resources, Supervision, Validation.

## References

[1] Hicks CW Selvin E. Epidemiology of peripheral neuropathy and lower extremity disease in diabetes. Curr Diabetes Rep. (2019) 19:86. doi: 10.1007/s11892-019-1212-8. PMC6755905 31456118

[2] Ziegler D Rathmann W Dickhaus T Meisinger C Mielck A KORA Study Group. Neuropathic pain in diabetes, prediabetes and normal glucose tolerance: the MONICA/KORA Augsburg Surveys S2 and S3. Pain Med. (2009) 10:393–400. doi: 10.1111/j.1526-4637.2008.00555.x. 19207236

[3] Galer BS Gianas A Jensen MP. Painful diabetic polyneuropathy: epidemiology, pain description, and quality of life. Diabetes Res Clin Pract. (2000) 47:123–8. doi: 10.1016/S0168-8227(99)00112-6. 10670912

[4] Jensen TS Karlsson P Gylfadottir SS Andersen ST Bennettn DL Tankisi H. Painful and non-painful diabetic neuropathy, diagnostic challenges and implications for future management. Brain. (2021) 144:1632–45. doi: 10.1093/brain/awab079. PMC8320269 33711103

[5] Feldman EL Callaghan BC Pop-Busui R Zochodne DW Wright DE Bennett DL. Diabetic neuropathy. Nat Rev Dis Primers. (2019) 5:41. doi: 10.1038/s41572-019-0092-1. 31197153

[6] Katsyuba E Auwerx J. ModulatingNAD+ metabolism, from bench to bedside. EMBO J. (2017) 36:2670–83. doi: 10.15252/embj.201797135. PMC5599801 28784597

[7] Yoshino J Mills KF Yoon MJ Imai S-I. Nicotinamide mononucleotide, a key NAD(+) intermediate, treats the pathophysiology of diet- and age-induced diabetes in mice. Cell Metab. (2011) 14:528–36. doi: 10.1016/j.cmet.2011.08.014. PMC3204926 21982712

[8] Verdin E. NAD(+) in aging, metabolism, and neurodegeneration. Science. (2015) 350:1208–13. doi: 10.1126/science.aac4854. 26785480

[9] Covarrubias AJ Perrone R Grozio A Verdin E. NAD(+) metabolism and its roles in cellular processes during ageing. Nat Rev Mol Cell Biol. (2021) 22:119–41. doi: 10.1038/s41580-020-00313-x. PMC7963035 33353981

[10] Fang EF Scheibye-Knudsen M Brace LE Kassahun SenGupta H Nilsen H. Defective mitophagy in XPA *via* PARP-1 hyperactivation and NAD(+)/SIRT1 reduction. Cell. (2014) 157:882–96. doi: 10.1016/j.cell.2014.03.026. PMC4625837 24813611

[11] Chandrasekaran K Muragundla A Demarest TG Choi J Sagi AR Najim N. mGluR2/3 activation of the SIRT1 axis preserves mitochondrial function in diabetic neuropathy. Ann Clin Transl Neurol. (2017) 4:844–58. doi: 10.1002/acn3.484. PMC5740254 29296613

[12] Chandrasekaran K Anjaneyulu M Choi J Kumar P Salimian M Ho C-Y. Role of mitochondria in diabetic peripheral neuropathy: Influencing the NAD(+)-dependent SIRT1-PGC-1alpha-TFAM pathway. Int Rev Neurobiol. (2019) 145:177–209. doi: 10.1016/bs.irn.2019.04.002. 31208524 PMC6590704

[13] Zhou B Wang DD Qiu Y Airhart S Liu Y Stempien-Otero A. Boosting NAD level suppresses inflammatory activation of PBMCs in heart failure. J Clin Invest. (2020) 130:6054–63. doi: 10.1172/JCI138538. PMC7598081 32790648

[14] Wang DD Airhart SE Zhou B Shireman LM Jiang S Rodriguez CM. Safety and tolerability of Nicotinamide riboside in heart failure with reduced ejection fraction. JACC Basic Transl Sci. (2022) 7:1183–96. doi: 10.1016/j.jacbts.2022.06.012. PMC9831861 36644285

[15] Feldman EL Nave KA Jensen TS Bennett DLH. New horizons in diabetic neuropathy: mechanisms, bioenergetics, and pain. Neuron. (2017) 93:1296–313. doi: 10.1016/j.neuron.2017.02.005. PMC5400015 28334605

[16] Hasin Y Seldin M Lusis A. Multi-omics approaches to disease. Genome Biol. (2017) 18:83. doi: 10.1186/s13059-017-1215-1. 28476144 PMC5418815

[17] Lin C Xu J-Q Zhong G-C Chen H Xue H-M Yang M. Integrating RNA-seq and scRNA-seq to explore the biological significance of NAD + metabolism-related genes in the initial diagnosis and relapse of childhood B-cell acute lymphoblastic leukemia. Front Immunol. (2022) 13:1043111. doi: 10.3389/fimmu.2022.1043111. 36439178 PMC9691973

[18] Ritchie ME Phipson B Wu D Hu Y Law CW Shi W. limma powers differential expression analyses for RNA-sequencing and microarray studies. Nucleic Acids Res. (2015) 43:e47. doi: 10.1093/nar/gkv007. 25605792 PMC4402510

[19] Yu G Wang LG Han Y He Q-Y. clusterProfiler: an R package for comparing biological themes among gene clusters. OMICS. (2012) 16:284–7. doi: 10.1089/omi.2011.0118. PMC3339379 22455463

[20] Conway JR Lex A Gehlenborg N. UpSetR: an R package for the visualization of intersecting sets and their properties. Bioinformatics. (2017) 33:2938–40. doi: 10.1093/bioinformatics/btx364. PMC5870712 28645171

[21] Sachs MC. plotROC: A tool for plotting ROC curves. J Stat Softw. (2017) 79. doi: 10.18637/jss.v079.c02. PMC6347406 30686944

[22] Rumora AE Savelieff MG Sakowski SA Feldma EL. Disorders of mitochondrial dynamics in peripheral neuropathy: Clues from hereditary neuropathy and diabetes. Int Rev Neurobiol. (2019) 145:127–76. doi: 10.1016/bs.irn.2019.05.002. PMC11533248 31208522

[23] Chandrasekaran K Najimi N Sagi AR Yarlagadda S Salimian M Arvas MI. NAD(+) precursors repair mitochondrial function in diabetes and prevent experimental diabetic neuropathy. Int J Mol Sci. (2022) 23. doi: 10.3390/ijms23094887. PMC9102948 35563288

[24] Waddell J Khatoon R Kristian T. Cellular and mitochondrial NAD homeostasis in health and disease. Cells. (2023) 12. doi: 10.3390/cells12091329. PMC10177113 37174729

[25] McLennan AG. The Nudix hydrolase superfamily. Cell Mol Life Sci. (2006) 63:123–43. doi: 10.1007/s00018-005-5386-7. PMC11136074 16378245

[26] Abdelraheim SR Spiller DG McLennan AG. Mammalian NADH diphosphatases of the Nudix family: cloning and characterization of the human peroxisomal NUDT12 protein. Biochem J. (2003) 374:329–35. doi: 10.1042/bj20030441. PMC1223609 12790796

[27] Mildvan AS Xia Z Azurmendi HF Saraswat V Legler PM Massiah MA. Structures and mechanisms of Nudix hydrolases. Arch Biochem Biophys. (2005) 433:129–43. doi: 10.1016/j.abb.2004.08.017. 15581572

[28] McLennan AG. Dinucleoside polyphosphates-friend or foe? Pharmacol Ther. (2000) 87:73–89. doi: 10.1016/S0163-7258(00)00041-3. 11007992

[29] Broome DT Pantalone KM Kashyap SR Philipson LH. Approach to the patient with MODY-monogenic diabetes. J Clin Endocrinol Metab. (2021) 106:237–50. doi: 10.1210/clinem/dgaa710. PMC7765647 33034350

[30] Kobayashi M Zochodne DW. Diabetic neuropathy and the sensory neuron: New aspects of pathogenesis and their treatment implications. J Diabetes Investig. (2018) 9:1239–54. doi: 10.1111/jdi.12833. PMC6215951 29533535

[31] Manu MS Rachana KS Advirao GM. Altered expression of IRS2 and GRB2 in demyelination of peripheral neurons: Implications in diabetic neuropathy. Neuropeptides. (2017) 62:71–9. doi: 10.1016/j.npep.2016.12.004. 28065675

[32] Stefan C Jansen S Bollen M. NPP-type ectophosphodiesterases: unity in diversity. Trends Biochem Sci. (2005) 30:542–50. doi: 10.1016/j.tibs.2005.08.005. 16125936

[33] Borza R Salgado-Polo F Moolenaar WH Perrakis A. Structure and function of the ecto-nucleotide pyrophosphatase/phosphodiesterase (ENPP) family: Tidying up diversity. J Biol Chem. (2022) 298:101526. doi: 10.1016/j.jbc.2021.101526. 34958798 PMC8808174

[34] Kawai T Akira S. The role of pattern-recognition receptors in innate immunity: update on Toll-like receptors. Nat Immunol. (2010) 11:373–84. doi: 10.1038/ni.1863. 20404851

[35] Nishida K Nomura Y Kawamori K Ohishi A Nagasawa K. ATP metabolizing enzymes ENPP1, 2 and 3 are localized in sensory neurons of rat dorsal root ganglion. Eur J Histochem. (2018) 62:2877. doi: 10.4081/ejh.2018.2877. 29943954 PMC6038112

[36] Aresta BM Gutierrez CA Dayton J Perrino BA Mutafova-Yambolieva VN. Mechanosensitive hydrolysis of ATP and ADP in lamina propria of the murine bladder by membrane-bound and soluble nucleotidases. Front Physiol. (2022) 13:918100. doi: 10.3389/fphys.2022.918100. 35784885 PMC9246094

[37] Yegutkin GG Boison D. ATP and adenosine metabolism in cancer: exploitation for therapeutic gain. Pharmacol Rev. (2022) 74:797–822. doi: 10.1124/pharmrev.121.000528. 35738682 PMC9553103

[38] Yegutkin GG. Enzymes involved in metabolism of extracellular nucleotides and nucleosides: functional implications and measurement of activities. Crit Rev Biochem Mol Biol. (2014) 49:473–97. doi: 10.3109/10409238.2014.953627. 25418535

[39] Park A Nam S. miRDM-rfGA: Genetic algorithm-based identification of a miRNA set for detecting type 2 diabetes. BMC Med Genomics. (2023) 16:195. doi: 10.1186/s12920-023-01636-2. 37608331 PMC10463588

[40] Brasnyo P Molnar GA Mohas M Markó L Laczy B Cseh J. Resveratrol improves insulin sensitivity, reduces oxidative stress and activates the Akt pathway in type 2 diabetic patients. Br J Nutr. (2011) 106:383–9. doi: 10.1017/S0007114511000316. 21385509

[41] Zhang W Yu H Lin Q Liu X Cheng Y Deng B. Anti-inflammatory effect of resveratrol attenuates the severity of diabetic neuropathy by activating the Nrf2 pathway. Aging (Albany NY). (2021) 13:10659–71. doi: 10.18632/aging.v13i7. PMC8064179 33770763

[42] Garcia-Martinez BI Ruiz-Ramos M Pedraza-Chaverri J Santiago-Osorio E Mendoza-Núñez VM. Effect of resveratrol on markers of oxidative stress and sirtuin 1 in elderly adults with type 2 diabetes. Int J Mol Sci. (2023) 24. doi: 10.3390/ijms24087422. PMC10138491 37108584

[43] Jeyaraman MM Al-Yousif NSH Singh Mann A Dolinsky VW Rabbani R Zarychanski R. Resveratrol for adults with type 2 diabetes mellitus. Cochrane Database Syst Rev. (2020) 1:CD011919. doi: 10.1002/14651858.CD011919.pub2. 31978258 PMC6984411

[44] Qiang X Satoh J Sagara M Fukuzawa M Masuda T Sakata Y. Inhibitory effect of troglitazone on diabetic neuropathy in streptozotocin-induced diabetic rats. Diabetologia. (1998) 41:1321–6. doi: 10.1007/s001250051072. 9833940

